# Supplementary material for: Modelling Skylarks (Alauda arvensis) to Predict Impacts of Changes in Land Management and Policy: Development and Testing of an Agent-Based Model
Source: PLoS One. 2013 Jun 6;8(6):e65803. doi: 10.1371/journal.pone.0065803 (PMC3675089; doi:10.1371/journal.pone.0065803)
Supplement: Supporting Information S4 — The skylark ODdox as a zipped archive. (ZIP) [file pone.0065803.s004.zip › Skylark_ODdox/dir_e33d76de6ce678176f3f982978eeda55.html]

ALMaSS Skylark ODdox: C:/CJT/MSVC/ALMaSS Working Source/BatchALMaSS/ Directory Reference


ALMaSS Skylark ODdox
 2.0


- Main Page
- Related Pages
- Classes
- Files

BatchALMaSS Directory Reference

|  |  |
| --- | --- |
| Files | |
| file | ALMaSS\_CmdLine.cpp |
| file | ALMaSS\_Setup.h |
| file | BinaryMapBase.cpp |
| file | BinaryMapBase.h |
| file | BoostRandomGenerators.h |
|  | **BoostRandomGenerators.h Boost headers for using the boost random number generation utilities** |
| file | MovementMap.cpp |
|  | **Movementmap.cpp This file contains the source for the MovementMap class** |
| file | MovementMap.h |
|  | **Movementmap.h This file contains the headers for the MovementMap class** |
| file | PopulationManager.cpp |
|  | **PopulationManager.cpp This is the code file for the population manager and associated classes** |
| file | populationmanager.h |
|  | **PopulationManager.h This is the header file for the population manager and associated classes** |
| file | PositionMap.cpp |
|  | **PositionMap.cpp This file contains the source for the PositionMap class** |
| file | positionmap.h |
|  | **PositionMap.h This file contains the headers for the PositionMap class** |
| file | References\_ODDox.h |


- CJT
- MSVC
- ALMaSS Working Source
- BatchALMaSS
- Generated on Thu Jan 10 2013 13:15:38 for ALMaSS Skylark ODdox by
   1.8.1.1
